# Pushing the DIEP Envelope: Where Are We Now?

**DOI:** 10.3390/jcm14176248

**Published:** 2025-09-04

**Authors:** Chase Clark, David A. Daar, Ara A. Salibian

**Affiliations:** 1 Division of Plastic and Reconstructive Surgery, Davis School of Medicine, University of California, Sacramento, CA 95817, USA; cecclark@ucdavis.edu; 2 Division of Plastic and Reconstructive Surgery, Keck Medicine of the University of Southern California, Los Angeles, CA 90033, USA; david.daar@med.usc.edu

**Keywords:** breast reconstruction, breast cancer, DIEP flap, conjoined flap, resensation, robotic surgery, microsurgery

## Abstract

The deep inferior epigastric perforator (DIEP) flap in breast reconstruction has been an evolution in providing an ideal autologous reconstruction while minimizing donor site morbidity. Innovations have continued to optimize the DIEP flap in multiple facets. Alternative flap designs, vasculature modifications, and conjoined and stacked flaps have improved the ability to increase flap volume and perfusion. Advancements in anatomic understanding of the abdomen have resulted in decreases in donor site morbidity and improved abdominal outcomes. Patient satisfaction regarding aesthetics has been enhanced through careful consideration of mastectomy techniques and recipient site modifications in addition to improved quality of life outcomes through sensory innervation. The study reviews the evolution and current state of abdominally-based breast reconstruction in its goal of optimizing aesthetic, patient-reported and quality-of-life outcomes while minimizing complications.

## 1. Introduction

Autologous tissue has been considered the gold standard for modern breast reconstruction in the appropriately indicated patient [1,2,3,4]. Microsurgical breast reconstruction has the ability to offer natural appearing results without many of the short- and long-term complications associated with implants including prosthesis infection, capsular contracture and rupture [5,6,7]. Additionally, patient reported outcomes via the validated BREAST-Q survey have demonstrated that patients who underwent autologous reconstruction have higher satisfaction rates compared to patients who underwent implant reconstruction [5,8].

Since the first description of the transverse rectus abdominus myocutaneous (TRAM) flap in 1979 [9,10], the abdomen has been the preferred site for autologous breast reconstruction. Today, the deep inferior epigastric perforator (DIEP) flap is the first choice for eligible patients [2,3,4,9,10] and comprised around 13% of all breast reconstructions in 2023 according to the American Society of Plastic Surgeons statistics [11]. Initial evolutions of this procedure included minimizing muscle harvest with the muscle-sparing TRAM (MS-TRAM) flap [9] followed by perforator-only techniques with the introduction of the DIEP flap [2,9,10].

The DIEP flap continues to be modified to improve different reconstruction facets. Alternative flap designs, combining flaps, and staging procedures attempt to enhance perfusion. Strides to decrease donor site morbidity include nerve sparing and minimizing muscle disruption. Aesthetic outcome is modified through selective mastectomy incisions and pre-reconstructive mastopexy. This narrative review summarizes the current advances in abdominally-based breast reconstruction with the goals of identifying modern standards of practice and future directions for improvement of care.

## 2. Discussion

### 2.1. Optimizing Volume and Perfusion

#### 2.1.1. Stacked and Conjoined DIEP Flaps

**Figure 1:**
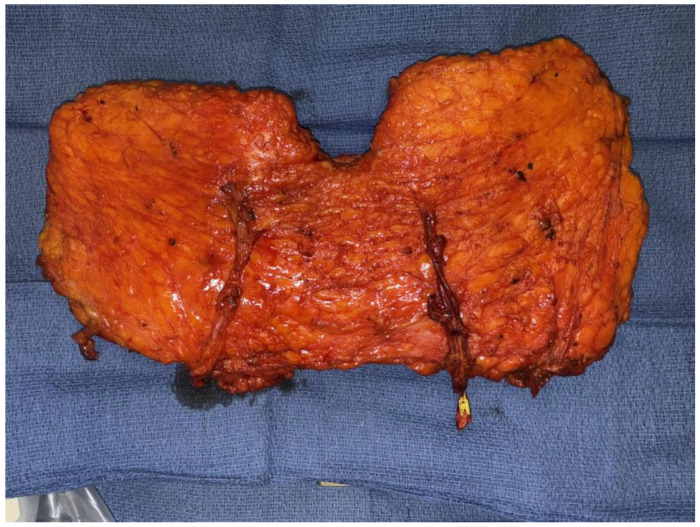
Bipedicled DIEP flap.

**Figure 2:**
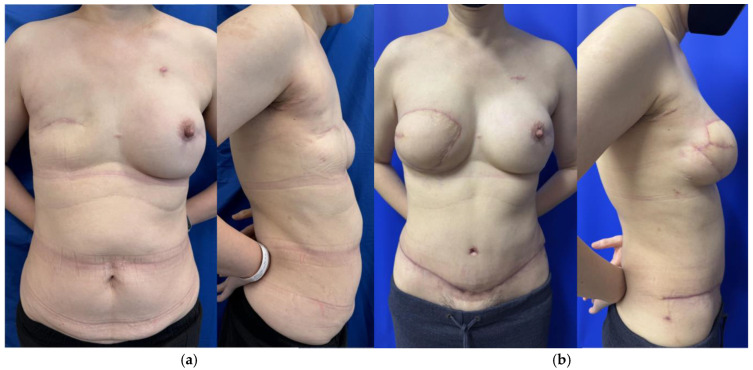
(**a**) Preoperative photo of patient after right mastectomy with no reconstruction and previous left nipple sparing mastectomy with implant reconstruction. (**b**) Same patient 3 months postop after right bipedicled DIEP flap reconstruction before revisions.

Patients who lack adequate donor tissue but desire autologous breast reconstruction may benefit from stacked- or conjoined-flap reconstructions. Stacked flaps involve more than one separate flap with each having its own distinct pedicle utilized for a single reconstruction, while a conjoined or bipedicled flap consists of a single piece of tissue perfused by multiple perforasomes based on more than one pedicle (Figure 1) [12,13]. The conjoined or bipedicled DIEP flap is most commonly utilized for a unilateral reconstruction when the entirety of the abdomen is needed to reconstruct the volume of a unilateral breast or when midline scarring may disrupt cross-midline perfusion (Figure 2) [12]. A meta-analysis of 26 studies analyzing stacked and conjoined flaps reported flap failure in 0.8% of flaps (N = 1952 flaps) and fat necrosis in 5.1% of flaps (N = 641 flaps) [14]. An additional retrospective cohort study demonstrated lower rates of flap necrosis in conjoined flaps compared to traditional extended hemi-abdominal flaps [10]. DIEP flaps can also be separated and “stacked” rather than maintaining midline continuity, with suggested benefits of improved projection and versatility in flap placement [15]. While stacked flaps may pose a challenge with regards to monitoring the buried flap, options include perfusing the buried flap via a branch of the primary flap or placing an implantable doppler.

In patients who require bilateral reconstruction, each hemiabdomen of a DIEP flap can be stacked with an additional flap for a four-flap breast reconstruction [16]. There are many different combinations of stacked flaps, but the most common flaps stacked with DIEP flaps are the profunda artery perforator (PAP) flaps. The shape of the PAP flap is complimentary to the inferior pole of the breast while the DIEP flap is complimentary to the superior pole, though this configuration may result in a scar across the middle of the reconstructed breast depending on the mastectomy incision [1,17]. Alternative flap designs to optimize scar placement have been described, including utilizing a conjoined DIEP flap on the side requiring more skin and stacked PAP flaps on the other [17]. The DIEP and lumbar artery perforator (LAP) stacked technique also allows for an enhanced aesthetic outcome through removing both abdominal and flank fat; however, this method requires position changes during the operation and additional vascular grafting to increase the length of the pedicle and match the diameter of the receiving vessels [1]. Other options for stacked flaps include combinations of transverse upper gracilis, MS-TRAM, and inferior or superior gluteal artery perforator flaps [10,17].

#### 2.1.2. Dual-Plane and Extended Flaps

Dual-Plane (DP) DIEP flaps utilize the superficial inferior epigastric artery (SIEA) and vein (SIEV) in addition to the deep inferior epigastric artery and vein to improve flap perfusion. The DP designation refers to the vascular design of the flap and should not be confused with the plane of placement; DP-flaps are pre-pectoral. Traditionally, the SIEV has been harvested in addition to the deep system to augment venous outflow in the case of flap congestion [12]. This important technique differs from DP-DIEP because the latter also uses the SIEA to increase inflow to the flap in cases of insufficient perfusion [12,18]. A retrospective cohort study comparing traditional DIEP flaps (N = 130) to DP-DIEP flaps (N = 114) demonstrated DP-DIEP flaps had lower rates of flap loss (2.5% in DIEP, 0% in DP-DIEP, *p* = 0.031) [19]. A prospective cohort study comparing traditional DIEP flaps (N = 35) to DP-DIEP flaps (N = 15) reported lower rates of fat necrosis among patients who underwent DP-DIEP reconstruction (14% in DIEP, 4% in DP-DIEP, *p* = 0.043) [18]. Use of the superficial abdominal vasculature to supercharge or turbocharge the deep system has the benefits of augmenting perfusion without additional donor site morbidity. While not necessary for all cases, this augmentation can be useful in situations with diminutive deep perforators or as a means harvesting less DIEP vessels to minimize donor site morbidity.

Extended flaps such as the stacked hemi-abdominal extended perforator (SHAEP) flap utilize a similar concept of added additional perforasomes based on nearby vessels to avoid the need for multiple donor sites as with traditional four-flap reconstructions [4,20]. The SHAEP flap design uses the traditional DIEP tissue but extends laterally on either side to include flank tissue [12,20]. The most common pedicle used in addition to the DIEP is the deep circumflex iliac perforator (DCIP), but other options include superficial circumflex iliac artery (SCIA), SIEA, lumbar arteries, or intercostal perforators [12,20]. The SHAEP flap also allows lateral “dog ears” to be utilized for reconstruction which are typically discarded in traditional DIEP flaps [20].

#### 2.1.3. Hybrid Autologous Techniques

Hybrid DIEP flap breast reconstruction involves the use of the DIEP flap in addition to an implant, mesh, or other materials to optimize breast volume in patients who do not have enough abdominal tissue for bilateral reconstructions. A DIEP-hybrid reconstruction allows for more conservative harvesting of the tissue and potentially a lower scar as well as obviates the need for additional donor sites for stacked flaps [16]. Hybrid reconstruction also optimizes the aesthetics of implant-based reconstruction by providing increased soft tissue coverage over a smaller implant, thereby minimizing implant visibility and rippling [16,21]. With regards to clinical outcomes, a comparison study of hybrid DIEP flap reconstruction versus implant only reconstruction demonstrated significantly less infection as well as higher efficacy of oral antibiotic treatment when infection arises [22]. Additionally, in patients who have had previous radiation treatment, hybrid flap reconstruction seems to reduce complications common to implant reconstruction alone, e.g., capsular contracture [23,24,25].

**Figure 3:**
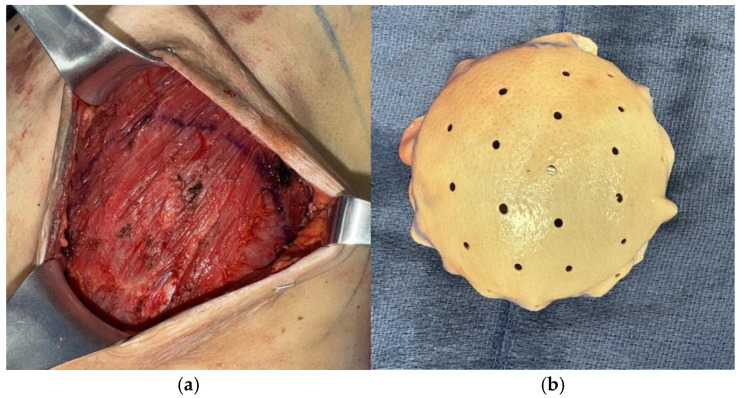

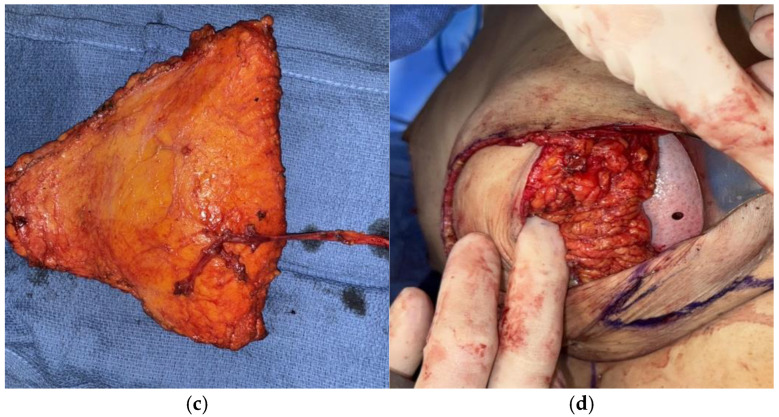
(**a**) Implant pocket design in prepectoral plane. (**b**) Implant sizer secured with anterior acellular dermal matrix. (**c**) DIEP flap harvest with elongated pedicle. (**d**) Lateral view of final implant secured within acellular dermal matrix placed onto the pectoralis major muscle after DIEP flap microsurgical anastomosis.

Various techniques for hybrid autologous reconstruction have been described. Traditionally implants have been placed in the subpectoral plane during hybrid reconstruction, however Momeni and Kanchwala have demonstrated safe practice with prepectoral plane insertion [26]. Immediate hybrid reconstruction involves the placement of the implant at the time of flap surgery. While concerns for immediate implant placement include compromising the flap pedicle, Kanchwala and Momeni describe using acellular dermal matrix (ADM) to secure implant position as well as harvesting a longer pedicle length to account for implant volume below the flap in a prepectoral position (Figure 3) [21].

Staged hybrid reconstruction can also be performed wherein the flap reconstruction is performed first and the implant is placed in a secondary surgery [23]. An advantage to delayed implant placement is correction of volume or projection asymmetry after tissues settle as well as maturation of the flap without necessarily relying on perfusion from the vascular pedicle [25]. The size needed to achieve the best result is often more easily determined when performed as a secondary procedure [23,25]. Disadvantages include increased technical challenges in forming the pocket for the implant [23]. A technique for pocket creation using ADM at time of flap reconstruction to allow easier implant placement at a later date has also been described [27].

Hybrid reconstructions with materials other than implants have also been described. HyPADTM breast reconstruction involves the use of acellular dermal matrix (ADM) folded to desired thickness and placed at the vertical meridian by the inframammary fold to add projection to the reconstructed breast [16]. Advantages of this technique are no long-term maintenance and none of the aesthetic considerations that would be present with implant reconstruction. Disadvantages include the high upfront cost of ADM [16]. Important patient considerations for this technique are that ADM can only add a small volume thus would not be suitable for patients who require significant volume [16].

#### 2.1.4. DIEP Delay

DIEP delay is a staged procedure wherein selected vessels are first optimized, then later harvested for reconstruction [4,28]. Indications for DIEP flap delay include the need for more tissue, prior liposuction or scarring, or inadequate perforators on preoperative imaging. The most common technique of delay involves making upper and lower incisions as well as a midline in a bilateral reconstruction, maintaining blood supply on a dominant perforator and a lateral skin bridge extending toward the flank [4]. A meta-analysis by Alves et al. reported delayed DIEP has similar outcomes to immediate reconstruction with equivalent rates of hematoma, infection, fat necrosis, and flap loss, though higher rates of wound healing complications [29]. Additionally, optimization of a single low lying perforator improves aesthetics through lower scar placement and may improve donor site morbidity [4,28]. While staging requires an additional surgery, in the appropriately selected patient DIEP delay enhances patient options and experience with little added morbidity [4].

### 2.2. Donor Site Optimization

#### 2.2.1. Nerve-Sparing DIEP

There are multiple studies assessing the pathways traveled by nerves throughout the rectus abdominis. Rozen et al. and Woodburne et al. reported nerves split into multiple branches prior to entering the rectus sheath lateral to the linea alba, whereas Gosling et al. and Duchateau et al. reported nerves enter the rectus abdominis on its posterior surface [2,30,31,32,33]. Stecco et al. detailed intercostal nerve trajectories in 10 abdominal walls from cadavers and reported that lateral deep inferior epigastric artery perforators tend to have significant nerve networks surrounding them [2].

While TRAM flaps lead to significantly higher abdominal hernia rates compared to DIEP flaps, MS2-TRAM flaps, which spare medial and lateral portions of the rectus muscle, have demonstrated no significant difference in abdominal wall bulge or hernia [34]. Additionally, motor nerve damage during DIEP harvest can cause weakness with abdominal flexion, which may be more common with harvest using lateral row perforators [2,35]. However, there is great variability in DIEP harvest techniques and their degree of muscle dissection, if any, which makes assessment of long-term donor site sequelae of DIEP flap and its comparisons to MS-TRAM flap difficult to assess [15,36].

Based on observation of nerve trajectories around lateral perforators, certain studies have recommended perforator selection at least 3 cm medial to the lateral edge of the rectus [2]. Rozen et al. similarly reported that medial perforators are more ideal to harvest given their decreased nerve association as observed through abdominal dissection of 20 cadavers [35]. Garvey et al. performed a large retrospective study looking at 615 flap donor sites over the course of ten years and compared donor site morbidity of medially versus laterally harvested flap demonstrating no significant difference in abdominal bulging or hernia across groups [37]. Their recommendation is to select perforators based on their quality and size instead of location [37]. Synthesizing recommendations from all studies, it would be reasonable to assess perforators based on their quality, size, and dominance, then upon observing no significant difference choosing a medial perforator to decrease denervation [2]. In this process, motor nerves traversing over the pedicle should be spared when possible, and if transection is necessary due to perforator locations repair can be performed.

#### 2.2.2. Minimally-Invasive DIEP Flap Harvest Techniques

Along similar lines of motor nerve preservation in the abdomen, many techniques have evolved to decrease donor site morbidity from DIEP flap harvest. In addition to nerve preservation, most of these techniques aim to minimize fascial incisions and muscle transection to minimize pain and decrease the risk of postoperative abdominal bulge.

#### 2.2.3. Limited Fascial Incision (LFI) Techniques

**Figure 4:**
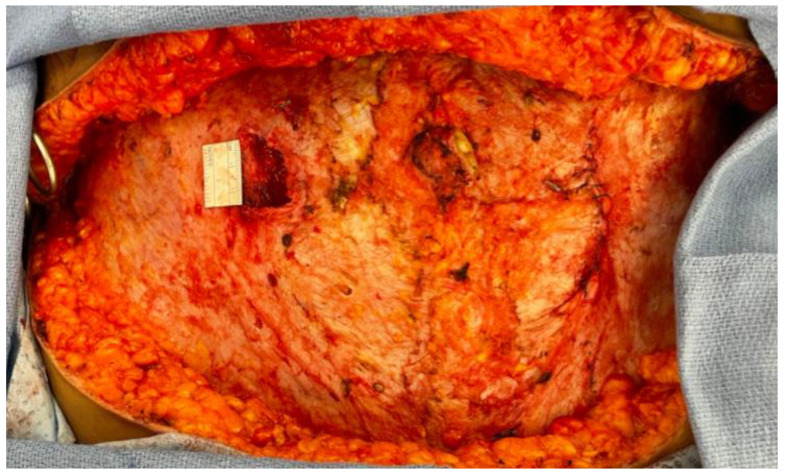
Limited fascial incision techniques (2.5 cm) can be performed without robotic or laparoscopic assistance, particularly with single-perforator flaps.

Harvesting the flap pedicle in an open approach can be done with limited fascial interruption through making short fascial incisions in the overlying muscle [38]. Typically this is performed by extending the fascial incision for the length of the intramuscular dissection of the perforator, and then tunneling to harvest the remainder of the retrorectus pedicle proximally. With a combination of good retraction and long instruments, this can routinely be performed to minimize fascial incisions (Figure 4). Hiven et al. compared DIEP flaps harvested in a traditional fashion to those harvested using LFI and demonstrated a greater difference between preoperative and postoperative EMG measurements in the traditional DIEP flap group resulting in less sensory side effects across the LFI group [38,39]. In a retrospective study looking at incision lengths, Martinez and Boutros reported an average of 1.7 cm (1.3–2.4 cm range) in single perforator flaps and 2.4 cm (2–2.5 cm range) in flaps with multiple perforators. Similarly, Hilven et al. report use of two incisions with lengths less than 4 cm and 3 cm in single perforator flaps [38]. Alternatively, Colohan et al. report a short and ultra-short pedicle DIEP flap design wherein the pedicle is dissected down to the lateral rectus edge to spare fascial incision lengthening [40]. Use of ultra-short pedicles can be more challenging given the potential for vessel mismatch and limited mobility of the pedicle for anastomosis. Use of LFI techniques optimize the donor site by preserving more muscle and expediting patient recovery [38,40,41].

#### 2.2.4. Laparoscopic Harvested DIEP Flap

Laparoscopic flap harvest has been advocated for to improve proximal pedicle visualization as well as ergonomics while trying to minimize fascial incision length [42,43]. A retrospective cohort study by Shakir et al. looking at 57 laparoscopically harvested DIEP flaps reported an average fascial length of 2 cm compared to 8–13 cm in the open approach which contributed to shorter hospital courses due to decreased need for narcotic pain control [43]. This study also reported an average operating time of 393 min which was 71 min longer than the open approach [43]. Abdominal site morbidity improves as fascial incision length decreases and laparoscopy allows surgeons to accomplish this while maintaining optimal body mechanics [42,43].

#### 2.2.5. Robotic DIEP Flap

A robotic approach for pedicle harvest has also been advocated for to minimize fascial incision [6,44]. In ideal candidates with a shorter intramuscular perforator course, this technique can limit abdominal wall fascial incision length, albeit with the need for intra-abdominal entry and insufflation [45]. Candidates for robotic harvested DIEP flaps are those with multiple perforators in close proximity as observed on pre-operative computed tomography angiography (CTA) [44,45]. The amount of muscle dissected is proportional to the distance between nonlinear perforators, thus the shorter the pathway the more muscle spared [44].

Bishop et al. presented a case study of 21 DIEP flaps harvested robotically which resulted in an average of 9.83 ± 2.28 cm of muscle spared compared to open approach [44]. However, it should be noted that non-robotic techniques with limited fascial incision have also reported short fascial incision lengths as discussed already in this narrative review [39,42]. Additionally, the robotic technique has challenges in the setting of prior intra-abdominal surgery, a steep learning curve, and requires additional operating time [6,44,46]. For bilateral reconstruction, Choi et al. and Bishop et al. reported an average console time of 65 ± 33 and 44.8 ± 9.3 min, respectively [44,46]. Comparison of total operating time by Choi et al. and Bishop et al. to the 322 min average time for open approach reported by Shakir et al. amounts to an additional 165 and 189 min, respectively [43,44,46]. Cost is also a critical factor and varies across institution policies, though there may be a potential gain in revenue for the hospital if it bills higher for robotic operations [44]. Robotic flap harvest may be the ideal choice in patients with short perforator distances in facilities with trained surgeons and readily available equipment.

#### 2.2.6. APEX Flap

**Figure 5:**
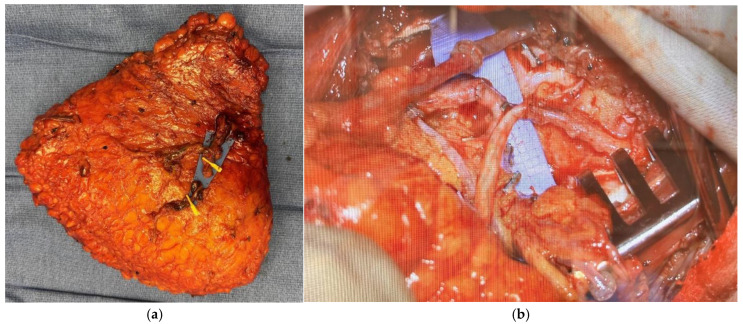
(**a**) APEX DIEP flap including high medial perforator, low lateral perforator, and background under location of divided perforator. (**b**) Final microanastomoses: DIEA/V to anterograde IMA/V to anterograde IMA/V (top left of photo), nerve coaptation (middle), and perforator re-anastomosis (bottom right).

When multiple perforators are deemed necessary from both the medial and lateral systems, a significant amount of muscle often has to be transected in a DIEP flap to allow for use of both vessels [9,15]. A technique known as abdominal perforator exchange (APEX) has allowed for use of multiple intramuscular perforators while sparing abdominal muscle completely and preserving motor nerves [9,15]. The APEX flap entails tracing disparate perforators down to their origin off the epigastric vessels, and then cutting and re-anastomosing the vessels above the muscle to prevent sacrifice of the intervening muscle (Figure 5). A retrospective study by Zoccali et al. reviewed 51 APEX flaps and reported no hernia or bulging in a 12 month follow up period with an average patient BMI of 32.1 ± 1.9 [9]. A retrospective study by DellaCroce et al. compared DIEP flaps (194) to APEX flaps (151) and discovered the latter spared an average of 2.6 cm of rectus muscle [37]. Zoccali et al. have recommended APEX flap reconstruction when more than one-third of the rectus muscle must be sacrificed or two or more motor nerve branches would be divided during pedicle isolation [9] (p. 163). Comparative cohort studies are needed to further elucidate the precise indications for when perforator exchange would decrease clinically-relevant abdominal wall morbidity.

#### 2.2.7. Mesh Reinforcement

Mesh has long been used to strengthen the abdominal wall in other surgical settings, but there is limited data on its use in DIEP flap reconstruction [47]. In a meta-analysis of 11 studies by Parmeshwar et al., an odds ratio of 0.28 was calculated for hernia occurrence in mesh closure of the DIEP flap donor site compared to primary closure, though it should be noted that no significant difference was reported across studies which reported only on bulging alone or bulging and hernia combined [48]. Additionally, the best material remains undetermined as biological meshes are more expensive while permanent meshes increase the risk for infection [47,48]. Wormer et al. evaluated use of a biosynthetic mesh compared to no mesh in a retrospective chart review and demonstrated no increase in complication rate and lower bulging rate in mesh containing groups over a 12 month follow up with an average BMI of 29.4 ± 4.4 [47]. More research is needed to determine the efficacy of mesh in preventing bulging and ideal material type after DIEP flap reconstruction.

### 2.3. Composite DIEP Flap

Vascularized lymph node transplant (VLNT) can be combined with DIEP flap breast reconstruction to treat breast cancer related lymphedema (BCRL) in a single procedure. The cluster of superficial abdominal lymph nodes along the SCIA allows for a composite lymph node flap to be easily harvested with the DIEP flap when needed [49,50]. Studies have demonstrated improved lymphedema outcomes in patients undergoing composite DIEP flaps as well as other outcome measures including decreased cellulitis frequency and compression regimes [51,52,53].

Considerations for composite DIEP design include pedicle orientation, lymph node orientation, and final flap placement [54]. Optimal placement of the transferred lymph nodes lacks consensus [55]. An important technique to minimize the risk of iatrogenic lower extremity lymphedema after groin VLNT is to perform reverse lymph node mapping in the ipsilateral extremity to harvest nodes draining the lower abdomen while avoiding lower extremity lymph nodes [55,56,57,58]. To address concerns about hollowing resulting from lymph node harvest, a strip of subcutaneous fat can be maintained in the upper abdominal flap and used to obliterate dead space [50,52,56]. Typically, when combined with a DIEP flap, the groin VLNT only requires additional anastomosis of a superficial vein in the axilla, though the SCIA can also be anastomosed if there is concern for hypoperfusion of the lymph node packet.

### 2.4. Optimizing Breast Aesthetics

#### 2.4.1. Mastectomy Flap Considerations

Mastectomy flap quality is the foundation for good reconstructive and aesthetic outcomes in breast reconstruction; therefore it is imperative that plastic surgeons and breast surgeons work collaboratively [59,60,61]. Key determinants of success include dissection technique, flap thickness and perfusion, and incision pattern [60,61]. Several flap dissection techniques exist, but critical factors for any dissection are preservation of the superficial subcutaneous tissue to avoid ischemic injury [60,62]. Flap thickness varies greatly with BMI and natural breast habitus and should therefore be considered individually in each patient [60,61].

Immediate autologous breast reconstruction has higher rates of mastectomy flap necrosis compared to two-stage prosthetic procedures, likely due to the increase pressure placed on skin envelopes as well as greater traction injury due to recipient vessel exposure and microsurgery [60,63]. However, mastectomy flap necrosis is influenced by many factors including tobacco, diabetes mellitus history, radiation therapy, high breast volume, large mastectomy weight, greater degree of ptosis, and most critical, mastectomy flap quality [60,61,62]. Staged approaches with tissue expander placement may help decrease this risk and were initially developed to avoid radiation damage to flaps in patients that required adjuvant therapy [64]. However, initial tissue expander placement also carries the risk of prosthesis complications, particularly in the setting of radiation, and additional surgery [65]. More recent studies have advocated for immediate autologous reconstruction even in the setting of anticipated adjuvant radiation therapy, citing minimal changes in flap volume and low complication rates [66]. In the end, these decisions should be individualized based on patient and surgeon preference as well as institutional radiation treatment protocols that can vary widely.

#### 2.4.2. Nipple Sparing Mastectomy Incisions

Nipple sparing mastectomy (NSM) has become a well-established technique for appropriately indicated patients to optimize aesthetics of the breast skin envelope [67,68,69,70]. The success of NSM in patients with appropriate breast morphology is highly dependent on preservation of perfusion the skin envelope and nipple areola complex (NAC) [59,62]. Staged DIEP breast reconstruction is particularly advantageous in the setting of nipple sparing mastectomies as it allows for mastectomy flap healing prior to the stress of flap inset and decreases nipple necrosis [28,71].

Multiple incisions have been described in nipple-sparing mastectomy. In a retrospective review published of different NSM incisions in immediate autologous reconstruction, patterns with the least amount of ischemic injury were vertical (5.8%) and radial lateral (7.8%) while the most were with Wise, or inverted T (36.1%), and inframammary (25%) [63]. While inframammary fold (IMF) incisions have reliably demonstrated low necrosis rates and good aesthetic outcomes in implant-based reconstruction [63,72,73,74], they have been associated with increased rates of skin envelope necrosis in immediate autologous reconstruction, potentially due to the increased traction to reach recipient vessels [63]. Periareolar incisions are generally avoided as they have been associated with significantly higher rates of nipple necrosis [60,61,63,75]. Deciding which pattern to use should be individualized to each patient based on their anatomy and goals with scar placement [74].

#### 2.4.3. Staged Reduction and Mastopexy

Patients with large or significantly ptotic breasts have increased risk for mastectomy flap and nipple necrosis, especially for nipple sparing techniques, due to increased perfusion demand and presence of a T-point with Wise pattern incisions [60,67,68,69,70]. Macromastia and ptosis also make tailoring the skin envelope after mastectomy challenging with regards to breast aesthetics. Staged mastopexy or reduction prior to NSM decreases mastectomy flap length and reduces redundancy, optimizes nipple placement, tightens the skin envelope, and improves aesthetic outcomes [59,60,67,68]. It is notable that with NSM, central position of the nipple can still be difficult to achieve with reports of greater than 30% of cases having nipple malposition [67].

Mastopexy/reduction performed in patients with large or ptotic breasts prior to mastectomy grants them the option of having a nipple sparing technique which might have otherwise carried too great a risk profile [59,67,68,69,70]. The best time frame between the mastopexy or reduction and the mastectomy is still uncertain. Too soon of an interval can lead to increased wound healing complications [70]. While some studies report no increased risks of wound healing between 10–12 weeks, others have recommended between 12–26 weeks for best aesthetic outcome [67,68,69]. It is recommended that the surgeon should intentionally leave the NAC slightly inferior during the mastopexy/reduction as it tends to lay more superiorly after breast reconstruction [69]. While staged mastopexy or reduction is safest in prophylactic mastectomies, there are risks to increasing the time to mastectomy in patients with cancer [69]. In non-prophylactic mastectomy patients, a partial mastectomy can be performed at the time of the initial oncoplastic mastopexy/reduction, followed by a completion mastectomy [68,69,70].

#### 2.4.4. Free Nipple Grafts

Patient reported outcomes in BREAST-Q demonstrate presence of a nipple-areola complex after reconstruction, whether it be via flap reconstruction, tattooing, free nipple grafts, or NAC preservation, improves satisfaction scores after autologous reconstruction [76]. Preservation of the NAC through NSM or free nipple grafts has higher satisfaction and aesthetic ratings compared to other reconstructive techniques [77].

**Figure 6:**
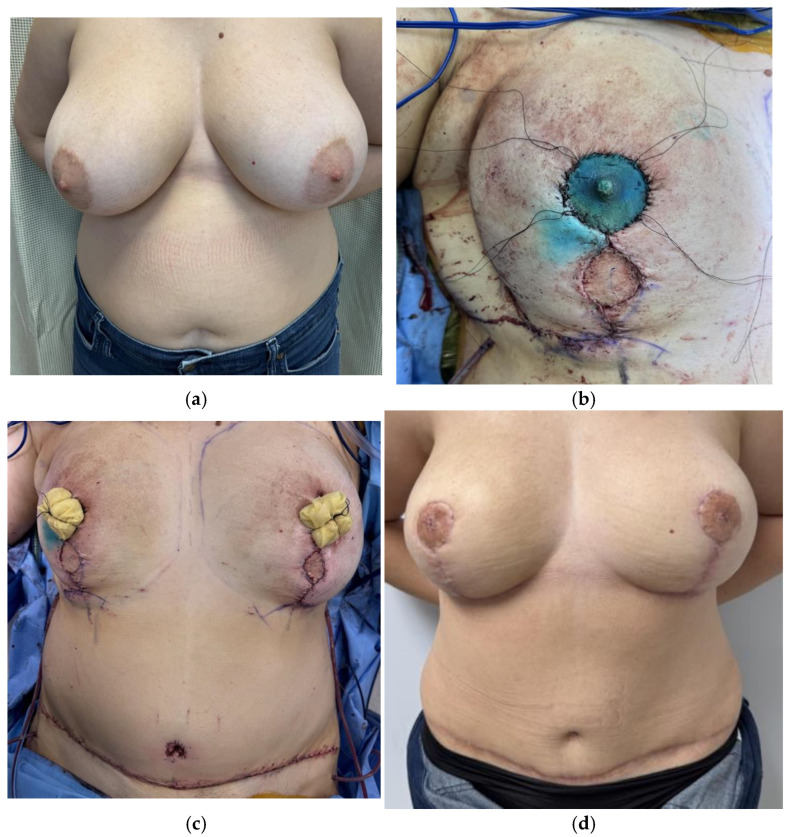
(**a**) Preoperative AP view of patient prior to mastectomy and DIEP flap reconstruction who is a candidate for nipple-sparing mastectomy due to large breasts and grade 2 ptosis. (**b**) Intraoperative photo of right DIEP flap inset with free nipple graft secured to flap and mastectomy skin edges. (**c**) Completion of bilateral DIEP flap and free nipple grafting with xeroform tie-over bolsters in place. (**d**) 14-month post-op from DIEP with free nipple grafting. The patient underwent 1 revision surgery including abdominal scar revision, bilateral mastopexy, and fat grafting.

Free nipple grafts are a good choice in patients who have large breasts, ptotic breasts, naturally asymmetric nipples, or cancer that is too close to the NAC to be eligible for NSM (Figure 6) [69,70]. The NAC can be harvested as a full thickness graft and frozen sections of the tissue just deep to the NAC sent to pathology for intraoperative guidance to ensure no cancerous tissue is kept [77,78]. Since the NAC needs to be thinned to facilitate healing, there can be loss of nipple projection [78]. The NAC is then inset onto a deepithelialized portion of the DIEP flap in the ideal position [77]. In a prospective cohort study looking at outcomes after free nipple grafts, Egan et al. reported that all patients experienced some degree of areolar discoloration and hypopigmentation, but this did not significantly affect satisfaction ratings [77].

### 2.5. Sensory Restoration

**Figure 7:**
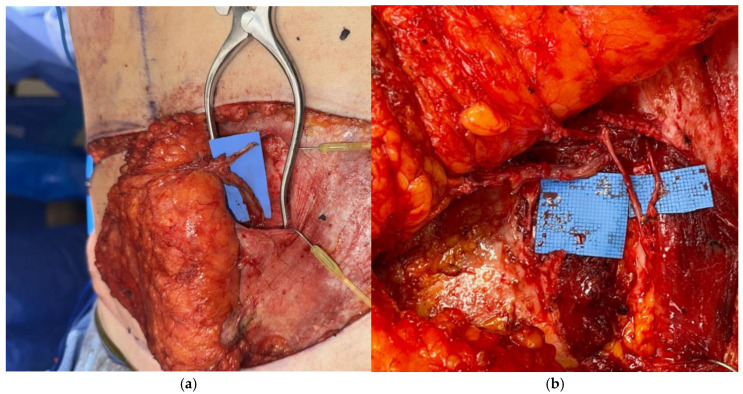
(**a**) DIEP flap harvest with inclusion of intercostal sensory nerve. (**b**) Motor nerves to the rectus abdominis (white arrows) are preserved whenever possible. If nerves are sacrificed (typically if in between two perforators), they can be easily repaired after flap harvest.

Loss of sensation after mastectomy has been demonstrated to have significant negative impacts on patient satisfaction and quality of life as well as increased risk of injury as a result of delayed response time [79,80,81,82,83]. Nerve coaptation for sensory restoration in autologous flap reconstruction was first performed in 1992 and later demonstrated possible with a DIEP flap in 1994 (Figure 7) [79,81]. There is mixed data regarding efficacy of type, degree, and timeline of sensory recovery after nerve restoration, with some studies finding enhanced results [79,80,81,83] while others report no difference [84].

Traditionally, the lateral cutaneous branch of the fourth intercostal nerve was used for flap neurotization; however, it often sustains iatrogenic injury during mastectomy or adds complexity to flap placement given its positioning [80]. Use of the anterior cutaneous branch of the third intercostal nerve as a recipient has also been promoted due to easier access given its location by the internal mammary vessels [80]. Multiple methods for nerve coaptation exist including direct coaptation, use of nerve graft, or use of a conduit. Use of a nerve conduit increases sensation recovery and better adapts to the mismatch in diameter of recipient and donor nerves [80]. Importantly, studies have demonstrated that harvesting of sensory nerves does not result in significant decreased sensation at donor site [79,81]. Multiple studies have also reported no significant increase in operative time for nerve coaptation [79,80,82].

## 3. Conclusions

From TRAM to MS-TRAM, to DIEP flap design, the optimal flap for breast reconstruction is constantly evolving. While the DIEP flap serves as the current gold standard for autologous breast reconstruction, advances in techniques further improve patient outcomes. In select patients, modifications to the DIEP flap design, combined flaps, and additional vascular supply can enhance flap viability. Multiple harvesting techniques to decrease and spare transected muscle and avoid nerve injury improve donor site morbidity. Additional aspects of patient satisfaction including aesthetic outcome and sensory recovery are bettered with careful selection of mastectomy pattern and flap innervation. The DIEP flap has come a long way since its introduction, yet continuous innovation and research further enhance standards of care and push the boundaries of current practices.

## Data Availability

No new data were created or analyzed in this study. Data sharing is not applicable to this article.
